# Evaluation of prototype of improved electron collimation system for Elekta linear accelerators

**DOI:** 10.1002/acm2.12342

**Published:** 2018-05-13

**Authors:** Garrett M. Pitcher, Kenneth R. Hogstrom, Robert L. Carver

**Affiliations:** ^1^ Department of Physics and Astronomy Louisiana State University Baton Rouge LA USA; ^2^ Mary Bird Perkins Cancer Center Baton Rouge LA USA

**Keywords:** electron collimation, electron Monte Carlo, radiation leakage dose, therapeutic electron beams

## Abstract

**Purpose:**

This study evaluated a new electron collimation system design for Elekta 6–20 MeV beams, which should reduce applicator weights by 25%–30%. Such reductions, as great as 3.9 kg for the largest applicator, should result in considerably easier handling by members of the radiotherapy team.

**Methods:**

Prototype 10 × 10 and 20 × 20‐cm^2^ applicators, used to measure weight, in‐field flatness, and out‐of‐field leakage dose, were constructed according to the previously published design with two minor modifications: (a) rather than tungsten, lead was used for trimmer material; and (b) continuous trimmer outer‐edge bevel was approximated by three steps. Because of lead plate softness, a 0.32‐cm aluminum plate replaced the equivalent lead thickness on the trimmer's downstream surface for structural support. Models of all applicators (6 × 6–25 × 25 cm^2^) with these modifications were inserted into a Monte Carlo (MC) model for dose calculations using 7, 13, and 20 MeV beams. Planar dose distributions were measured and calculated at 1‐ and 2‐cm water depths to evaluate in‐field beam flatness and out‐of‐field leakage dose.

**Results:**

Prototype 10 × 10 and 20 × 20‐cm^2^ applicator measurements agreed with calculated weights, in‐field flatness, and out‐of‐field leakage doses for 7, 13, and 20 MeV beams. Also, MC dose calculations showed that all applicators (6 × 6–25 × 25 cm^2^) and 7, 13, and 20 MeV beams met our stringent in‐field flatness specifications (±3% major axes; ±4% diagonals) and IEC out‐of‐field leakage dose specifications.

**Conclusions:**

Our results validated the new electron collimating system design for Elekta 6–20 MeV electron beams, which could serve as basis for a new clinical electron collimating system with significantly reduced applicator weights.

## 1 INTRODUCTION

Our cancer clinic has seven Elekta Infinity radiotherapy accelerators (MLCi2 treatment head) with matched, custom electron beams spanning 7–20 MeV (R_90_ values of 2.0, 2.5, 3.0, 3.5, 4.0, 5.0, and 6.0 ± 0.1 cm) and slightly modified scattering foils.^1^, ^2^ Although they have exceptional in‐field flatness laterally (±3% along major axes; ±4% along diagonals^3^) and out‐of‐field leakage doses well below IEC standards^4^ for all applicators (6 × 6–25 × 25 cm^2^), there is opportunity to improve the delivery technology by reducing the weight of the electron applicators, particularly those for larger fields.^5^, ^6^ To that end, an optimization procedure utilizing analytical pencil beam and Monte Carlo (MC) calculations was developed to design significantly lighter applicators with similar in‐field flatness and out‐of‐field leakage dose as the current ones. Results showed significantly reduced applicator trimmer weights, which should translate to applicator weights for 6 × 6, 10 × 10, 14 × 14, 20 × 20, and 25 × 25‐cm^2^ applicators for 6–20 MeV beams being reduced by 7.0→5.1, 7.7→5.8, 9.1→6.7, 10.9→7.6, and 13.4→9.5 kg, respectively.^5^ Such reductions should result in considerably easier handling by members of the radiotherapy team.

The purpose of this study was to validate these designs. First, prototype 10 × 10 and 20 × 20‐cm^2^ applicators were constructed and used to measure weight, in‐field flatness, and out‐of‐field leakage dose. The two applicators were constructed according to previously published design^5^ with two minor modifications: (a) lead was substituted for tungsten for trimmer material, which required a 0.32‐cm aluminum plate replacing the equivalent lead thickness on the trimmer's downstream surface for structural support, and (b) trimmer outer‐edge bevel was approximated by three steps. Second, all five applicators were modeled with the two minor modifications in a MC code for calculation of in‐field flatness and out‐of‐field leakage doses. Results will show that both measurements and calculations exhibited expected weights, in‐field flatness, and out‐of‐field leakage doses.

## 2 METHODS

Methods used to evaluate the new, lighter electron applicators designed and previously reported by Pitcher et al. 5 are described. Prototype 10 × 10 and 20 × 20‐cm^2^ electron applicators with two minor modifications were fabricated and used for dose measurements. Models of all applicators (6 × 6, 10 × 10, 14 × 14, 20 × 20, and 25 × 25‐cm^2^) with the two minor modifications were inserted into a Monte Carlo (MC) calculation for dose calculations using 7, 13, and 20 MeV beams.

### 2.A Designs for new, lighter applicators

Pitcher et al. 5 outlined a design procedure for a full set of applicators (6 × 6–25 × 25 cm^2^) for 6–20 MeV beams. Its 10 × 10 and 20 × 20‐cm^2^ applicator designs (trimmers and x‐ray jaw settings) provided the basis for a new Elekta electron collimation system with lighter applicators, including 6 × 6, 14 × 14, and 25 × 25‐cm^2^ applicators. All new applicator designs retained the design parameters from the 10 × 10 and 20 × 20‐cm^2^ applicators (i.e., trimmer positions, materials, inner‐edge divergence angles, outer‐edge bevel forming fluence matching off‐axis ratios (OARs), and trimmer thicknesses) except for OARs at each trimmers inner edge, as calculated using a pencil beam algorithm. The 6 × 6 and 25 × 25‐cm^2^ applicators used the same inner‐edge OARs as the 10 × 10 and 20 × 20‐cm^2^ applicators, respectively, for a 6‐MeV beam. The 14 × 14‐cm^2^ applicator inner‐edge OARs were linearly interpolated between the 10 × 10 and 20 × 20‐cm^2^ OARs for a 6‐MeV beam. x‐ray jaw positions for each energy were determined by linearly interpolating the OARs at the upper trimmer inner edge between the 6‐MeV value and 55% at 20 MeV for all applicators (c.f. Table 1).

**Table 1:** Trimmer inner‐edge fluence matching OARs for the design of each prototype applicator

	6 × 6 cm^2^	10 × 10 cm^2^	14 × 14 cm^2^	20 × 20 cm^2^	25 × 25 cm^2^
Lower trimmer inner‐edge matching OAR at 6 MeV	96.5%	96.5%	95.0%	93.0%	93.0%
Middle trimmer inner‐edge matching OAR at 6 MeV	96.0%	96.0%	94.0%	91.0%	91.0%
Upper trimmer inner‐edge matching OAR at 6 MeV	95.0%	95.0%	92.5%	89.0%	89.0%
Upper trimmer inner‐edge matching OAR at 20 MeV	55.0%	55.0%	55.0%	55.0%	55.0%

### 2.B Fabrication of 10 × 10 and 20 × 20‐cm^2^ prototype applicators

Prototype 10 × 10 and 20 × 20‐cm^2^ applicators were fabricated by the Louisiana State University (LSU) Physics and Astronomy machine shop according to the new design specifications summarized above with two minor modifications. First, the prototype trimmers were milled from lead rather than tungsten because of the difficulty in milling tungsten in our shop. Second, the smooth, beveled shape of the trimmer outer edge was approximated by three steps due to the shop not possessing the equipment required to mill the curved shape.

Previous MC studies of electron transmission in lead and tungsten showed that the thicknesses to stop 99% of the electrons in a 20 MeV beam were close, 9.08 and 9.56 g cm^−2^, respectively.^6^ Although lead is more effective at stopping electrons, it requires a greater thickness due to their densities of 11.3 and 19.0 g cm^−3^, respectively. Because of the softness of lead plate the trimmers were backed with a 0.32‐cm aluminum plate (2.7 g cm^−3^) for structural support. The aluminum plate was placed against the downstream surface of the lead trimmer, spanning the full trimmer width; although previous investigators have shown the benefit of placing aluminum on the upstream surface,^7^ our goal was to best simulate the tungsten design. The lead plate's thickness (g cm^−2^) was reduced by 9%, the added thickness (g cm^−2^) of the aluminum plate for the middle and lower trimmers. This same reduction was not made for the upper trimmer, as the thickness of this trimmer had already been reduced,^5^ and any further reduction in its thickness would cause difficulties in machining the lead.

**Figure 1:**
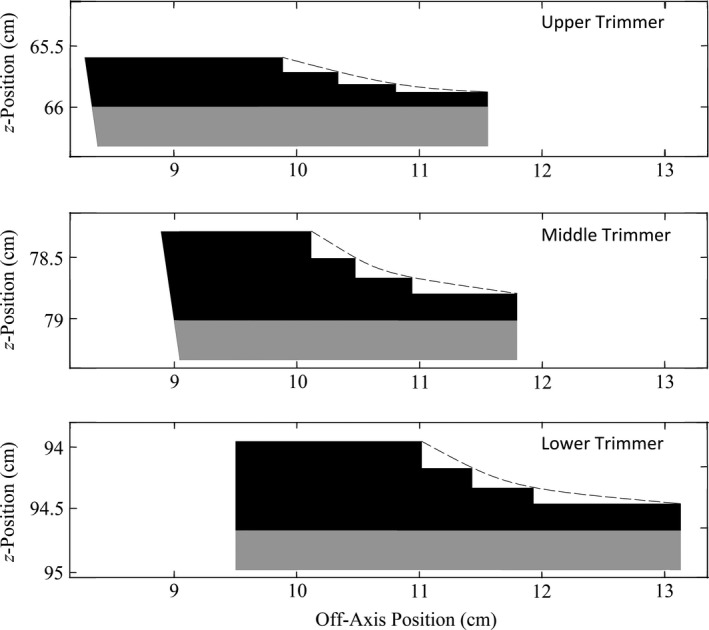
In‐plane cross‐sectional views of the upper, middle, and lower trimmers designed with modifications for fabrication. The black and gray regions demarcate the lead and aluminum materials, respectively, of the 20 × 20‐cm^2^ prototype in the in‐plane dimension, which differs from the cross‐plane dimension only in width of the upper trimmer^5^, ^6^. The vertical and horizontal axes (isocenter at z = 100 cm; central axis at off‐axis position = 0) indicate the *z* position and off‐axis position, respectively, of each trimmer. The stepped approximation to the smooth bevel (dashed line) can be seen along the outer edge of the lead trimmer component. The divergence of the upper and middle trimmer inner edges backproject to approximately z = 0^5^, ^6^.

Figure 1 illustrates this configuration, showing a cross‐sectional view of the prototype 20 × 20‐cm^2^ designs of the upper, middle, and lower trimmers. For the upper two trimmers, the inner edges were designed divergent to align with the beam's divergence, while the lower trimmer inner edges were designed parallel to central axis.

To fabricate using the available machining equipment, the outer‐edge bevel was approximated by a series of steps, rather than a smooth curve. Four discrete energies were selected to match the width and thickness of each step: 6, 9, 13, and 20 MeV. The outer edge of each step matched the off‐axis position of the specified fluence OAR for the associated beam energy. The thickness of each step was calculated with the same electron energy using the range energy curve developed from the MC 1% threshold analysis.^6^ The effect of this modification to the outer‐edge bevel can be seen in Fig. 1.

**Figure 2:**
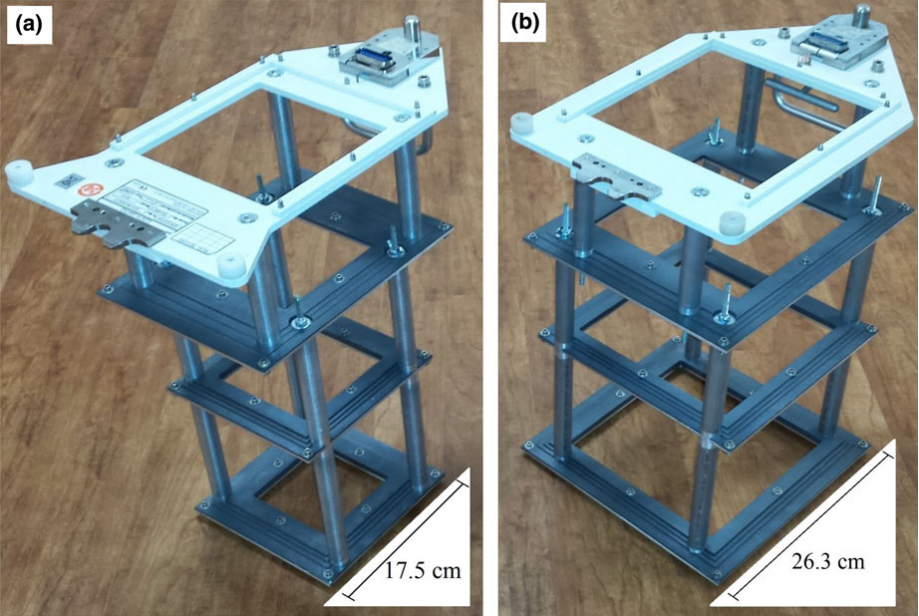
View of prototype applicators: (a) 10 × 10 and (b) 20 × 20 cm^2^. Downstream of the white plate mounting the applicator to the Elekta treatment head are the upper, middle, and lower trimmer bars separated by aluminum tubing and held together by threaded rods. Note the stepped outer edges of each lead trimmer and the thin downstream aluminum plates.

The resulting, fabricated 10 × 10 and 20 × 20‐cm^2^ prototype applicators are shown in Figs. 2(a) and 2(b), respectively. The trimmers and attachment plate were separated at appropriate distances from one another using a set of aluminum spacer tubes, which can be seen in Fig. 2 near the corners of each trimmer. Threaded rods were inserted inside these spacing tubes and nuts were tightened on each end to rigidly hold the entire assembly of trimmers and spacing tubes together.

### 2.C MC‐calculated dose distributions in patient plane for prototype applicators

Each of the designed prototype applicators (6 × 6–25 × 25 cm^2^) with the minor modifications described above were modeled in BEAMnrc to allow MC calculations of the underlying dose at low, medium, and high energies (7, 13, and 20 MeV). These dose calculations provided data used to evaluate in‐field beam flatness and out‐of‐field leakage dose.

Each of these five applicator designs was modeled in BEAMnrc by inserting them into a previously verified model of our Elekta Infinity accelerator (MLCi2 treatment head).^1^, ^2^ Simulations were performed with these prototype collimation systems with the 7 MeV (E_p,0_ = 7.14 MeV, R_90_ = 2.0 cm), 13 MeV (E_p,0_ = 13.12 MeV, R_90_ = 4.0 cm), and 20 MeV (E_p,0_ = 20.47 MeV, R_90_ = 6.0 cm) beams to score phase space files 1 cm upstream of isocenter. These phase spaces were used as input to DOSXYZnrc for dose calculation, in which the phase space particles were transported through 1 cm of air into a water phantom at 100‐cm SSD. Dose distributions were calculated in horizontal matrices of 0.5 × 0.5 × 0.5‐cm^3^ water voxels centered at 1 cm (ranging from 0.75 to 1.25 cm) and 2 cm (ranging from 1.75 to 2.25 cm) depth in water. The distributions were symmetrized in both the in‐plane and cross‐plane dimensions by reflecting the distributions about central axis to reduce statistical uncertainty, and all profiles were normalized to the mean dose calculated within a 3 × 3 set of voxels centered about central axis at the calculation depth.

### 2.D MC lateral leakage analysis

In addition to the specifications for maximum and mean leakage dose at the patient plane, the IEC^4^ specifies that the leakage along the vertical sides of the applicator at a position 2 cm outside the volume contained by the applicator not exceed 10% of D_max_ (maximum dose in water on central axis at 100‐cm SSD).

To investigate the leakage dose along the sides of the applicator, a MC investigation was performed with the prototype 10 × 10 and 20 × 20‐cm^2^ applicators in the Elekta Infinity BEAMnrc model. Simulations were performed with the 7, 13, and 20 MeV beams and phase space data were scored at three different horizontal planes with *z* positions of 79, 88, and 95 cm. These simulations maintained all source and transport parameters from the previous studies using the BEAMnrc model.

Using these phase space files as source input, DOSXYZnrc was used to calculate the horizontal leakage dose profiles along the side of the applicator. Particles (10^9^ histories) were transported through 1 cm of air and dose profiles were calculated in 0.5 × 0.5 × 0.5‐cm^3^ voxels centered at 1‐cm depth in water, such that the *z* positions of the three dose calculation planes were 81, 90, and 97 cm. The DOSXYZnrc water phantoms used for these calculations were identical to those used in the other MC studies in this project, except that they were situated at different *z* positions. To reduce statistical uncertainty, the dose distributions were symmetrized in both the in‐plane and cross‐plane dimensions by reflecting them about central axis. Each profile was normalized to D_max_ in a water phantom at 100‐cm SSD for each beam energy. The calculated dose profiles outside the volume contained by the applicator were analyzed to evaluate the leakage dose along the side of the applicator.

### 2.E Measurement of in‐field flatness and out‐of‐field leakage dose

A set of off‐axis dose measurements was performed to evaluate the prototype collimation system for both the in‐field beam flatness and out‐of‐field leakage dose at the patient plane with the prototype 10 × 10 and 20 × 20‐cm^2^ applicators. The prototype applicators were attached to the Elekta Infinity accelerator with the beam aimed vertically downward. The jaws were positioned in their designated locations within the prototype collimation system design for each beam energy. Off‐axis profiles were measured in the in‐plane, cross‐plane, and both diagonal directions using an OmniPro two‐dimensional scanning tank with OmniPro (v. 6.2) scanning software package (Scanditronix Wellhofer AB RFA 20‐SERVO, Uppsala, Sweden) at depths of 1 and 2 cm in water. Two N31011, 0.125 cm^3^ cylindrical ionization chambers (PTW, Freiburg, Germany) were used for measurement, one for scanning and one for reference. The scanning chamber was oriented such that the stem was perpendicular to central axis of the beam and to the direction of the scan. The reference chamber was attached to the applicator in the corner of the field. For the diagonal scans, the reference chamber was moved to a field corner not coinciding with the scanned profile. The beam scanning system was rotated 0°, 90°, and ±45° by rotating the treatment couch to measure the in‐plane, cross‐plane, and diagonal profiles, respectively.

Each profile was normalized to the central‐axis ionization value at the depth of the scan. The profiles were then centered by shifting them such that the 50% ionization levels were equal distances from the central axis. For evaluating the in‐field results, the profiles were symmetrized about central axis by averaging the measurement values at each negative and positive off‐axis position. No symmetrization was performed for evaluating the out‐of‐field results. Relative dose was assumed equal to relative ionization, i.e., conversion factors from ionization to dose were assumed identical for central‐axis and off‐axis positions at equal depths. These measurements were used to compare the leakage dose of the prototype collimation system with those of the current clinical Elekta applicators.

In‐field flatness was evaluated according to the criteria described by Hogstrom,^3^ which states that off‐axis dose vary from the central‐axis dose by no more than ±3% along the major axes (in‐plane and cross‐plane) and ±4% along the diagonal axes. These specifications are assessed at 1 cm depth in water for E_p,0_ ≤ 9 MeV and 2 cm for E_p,0_ > 9 MeV within a region 2 cm inside the field edge for the major axes and 2$\sqrt{2}$ cm for the diagonal axes.^1^, ^3^

The out‐of‐field leakage dose was evaluated according to criteria described by the IEC,^4^ which specifies that the maximum leakage dose not exceed 10% of D_max_ and mean leakage dose not exceed a value between 1% and 1.8% of D_max_, which varies with beam energy. The beams used in this study, 7, 13, and 20 MeV, had specified mean leakage limits of 1.00%, 1.10%, and 1.34%, respectively (determined using E_p,0_ values of 7.14, 13.12, and 20.47 MeV, respectively). Consistent with IEC specifications, mean and maximum leakage doses were calculated from measured dose (relative dose divided by central‐axis percent of D_max_, i.e., 95.2%, 94.5%, and 97.8%, respectively) points sampled at 2 cm spacing along the in‐plane, cross‐plane, and diagonal axes at 1 cm depth in water at 100 cm SSD. These sampled points spanned from an inner boundary located 2 and 4 cm outside the field edge for maximum and mean dose determination, respectively, out to the geometric projection of the primary collimator at isocenter, located 24.8 cm off‐axis for the Elekta infinity accelerator.^1^, ^4^

## 3 RESULTS AND DISCUSSION

### 3.A Applicator and trimmer weights for newly designed models (6 × 6–25 × 25 cm^2^) and fabricated prototypes (10 × 10 and 20 × 20 cm^2^)

The two minor modifications to the Pitcher et al. applicator models, required for machining purposes, slightly decreased their trimmer weights, from 3.73 to 3.66 kg for the 10 × 10‐cm^2^ applicator and from 5.09 to 4.95 kg for the 20 × 20‐cm^2^ applicator. These slight weight reductions were attributed to two factors. First, the range (in g cm^−2^) of a 20 MeV electron beam calculated using the MC 1% threshold method equation is slightly less for lead than tungsten.^6^ Second, by approximating the outer edge as a series of steps, a small amount of material was removed from the designed bevel shape (c.f. Fig. 1).

Once assembled, the full weight (trimmers plus all structural materials, such as the attachment plate, aluminum spacer tubes, and threaded rods) of the prototype 10 × 10 and 20 × 20‐cm^2^ applicators was measured to be 5.5 and 6.8 kg, respectively. These values do not include the weight of the various components (e.g., the locking mechanism for the field defining inserts and the electronic components associated with the collisional interlocking system), which are present on the current Elekta applicators but not on the fabricated prototype. The full applicator weights of the new design were estimated by adding the difference in the full applicator and trimmer weights of the current Elekta applicators to the trimmer weights of the prototype design. This calculation estimated the full weight of the new 10 × 10 and 20 × 20‐cm^2^ applicator designs to be 5.8 and 7.6 kg, respectively, compared with 7.7 and 10.9 kg for the current Elekta applicators.

These weight results are listed in Table 2, which compares the total trimmer weights and full applicator weights of the new design and the current clinical Elekta and Varian applicators for all applicator sizes. The results show that the prototype models surpassed both the current clinical Elekta and Varian designs for all sizes for both total trimmer weight and full applicator weight.

**Table 2:** Comparison of applicator weights in kg of both trimmers only and full applicator for current Elekta, current Varian, and prototype Elekta electron collimating systems for each applicator size (6 × 6–25 × 25 cm^2^)

Applicator size (cm^2^)	Current Elekta		Current Varian		Prototype Elekta		
	Trimmers only	Full applicator	Trimmers only	Full applicator	Trimmers only	Full applicator	Measured prototype
6 × 6	4.75	7.0	4.20^b^	5.7	2.79	5.1^c^	–
10 × 10	5.52	7.7	5.00^b^	6.5	3.66	5.8^c^	5.5^d^
14 × 14	6.71	9.1	6.10^a^, ^b^	7.6^a^	4.15	6.7^c^	–
20 × 20	8.36	10.9	7.10^b^	8.6	4.95	7.6^c^	6.8^d^
25 × 25	10.00	13.4	8.00^b^	9.5	5.91	9.5^c^	–

a Weights are for 15 × 15‐cm^2^ applicator.

b Varian trimmer weights were estimated as full applicator weight minus 1.5 kg.

c Prototype Elekta full applicator weights were estimated as the prototype trimmer weight, plus the difference in the current Elekta full applicator and trimmer weights.

d Measured Prototype weights were measured by weighing the fabricated prototypes. The differences from the Full applicator weights are attributed to the electrical and structural components of the Current Elekta applicators’ collision interlocking mechanisms not being present on the fabricated prototype design.

### 3.B In‐field beam flatness at 100‐cm SSD

**Figure 3:**
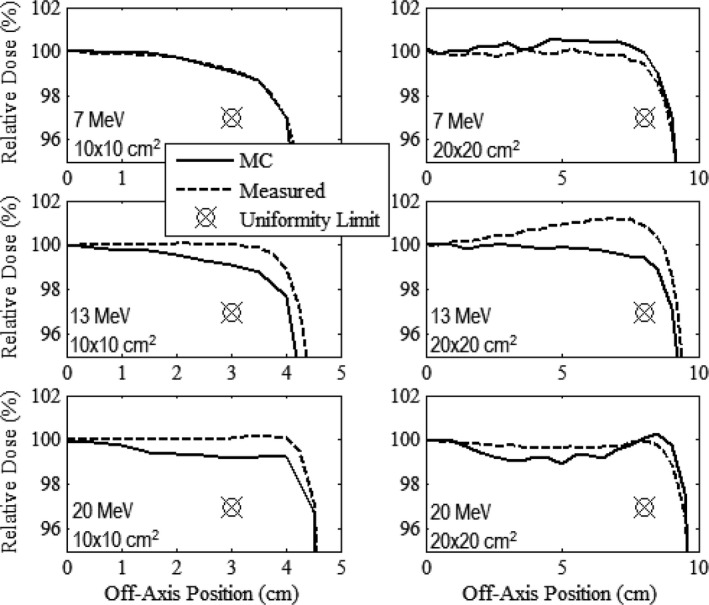
Comparison of measured and MC calculated in‐plane profiles of relative dose versus off‐axis position in water (100‐cm SSD) for the prototype 10 × 10 (left column) and 20 × 20‐cm^2^ (right column) applicators at beam energies of 7 (upper row), 13 (middle row), and 20 MeV (lower row). Profiles are normalized to central‐axis dose at the measurement depth; 7‐MeV profiles are at 1‐cm depth in water; 13 and 20‐MeV profiles are at 2‐cm depth. The uniformity limit marker (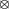) represents the minimum dose at the edge of the uniformity region required to pass our flatness criteria.

**Figure 4:**
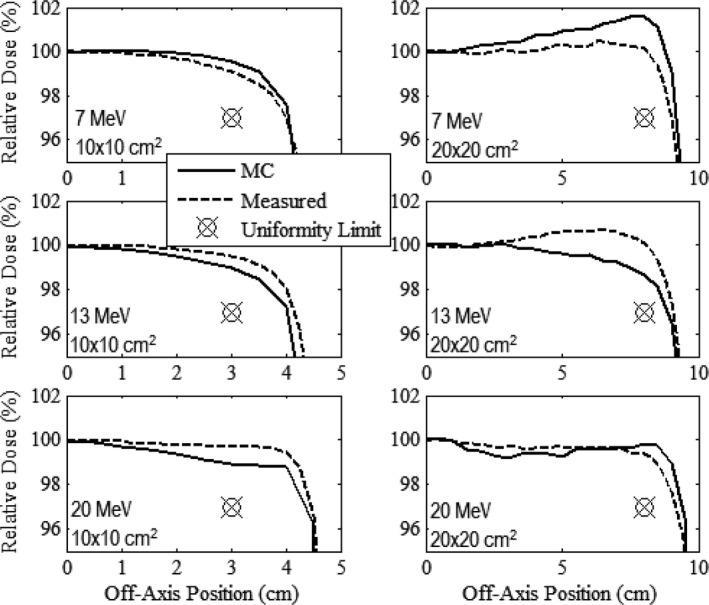
Comparison of measured and MC‐calculated cross‐plane profiles of relative dose versus off‐axis position in water (100‐cm SSD) with the same comparisons and measurement conditions as Fig. 3.

**Figure 5:**
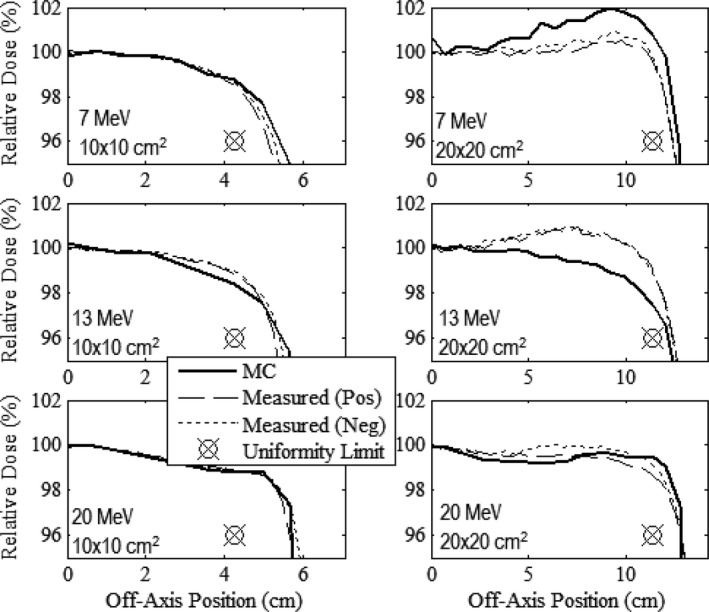
Comparison of measured and MC‐calculated diagonal profiles of relative dose versus off‐axis position in water (100‐cm SSD) with the same comparisons and measurement conditions as Fig. 3. The positive diagonal measurement profiles (long dashed) were scanned diagonally from the negative in‐plane (toward gantry) and negative cross‐plane (to the left when facing the gantry) quadrant to the positive in‐plane (away from gantry) and positive cross‐plane (to the right when facing the gantry) quadrant. The negative diagonal measurement profiles (small dashed) were scanned diagonally from the positive in‐plane and negative cross‐plane quadrant, to the negative in‐plane and positive cross‐plane quadrant.

Figures 3, 4, 5 compare measured and MC‐calculated in‐field plots of relative dose versus off‐axis position for the in‐plane, cross‐plane, and diagonal axes, respectively. Each figure contains six plots, all combinations of the 10 × 10 and 20 × 20‐cm^2^ prototype collimation systems with the 7, 13, and 20 MeV beams. The 7 MeV beam profiles were measured at 1‐cm depth in water, and the 13 and 20 MeV beam profiles were measured at 2‐cm depth.

Relative dose profiles were deemed to be acceptably flat if they fell within 100 ± 3% (major axes) or 100 ± 4% (diagonal axes) between central axis and the uniformity limit marker located 2 cm inside the edge of the field for the major axes and 2$\sqrt{2}$ cm for the diagonals. Figures 3, 4, 5 show that all dose profiles, both measured and calculated, meet this criteria, confirming that both the 10 × 10 and 20 × 20‐cm^2^ collimation systems produced acceptably flat beams.

When comparing the MC‐calculated and measured dose profiles, the plots show that the MC calculations overpredicted the measured dose near the edge of the field by as much as 1.5% for the 7 MeV profiles, and underpredicted the measured dose near the edge of the field by as much as 1.6% for the 13 MeV profiles. For the 20 MeV profiles, the MC calculations in general slightly underpredicted the measured dose within approximately 7 cm of central axis, but slightly overpredicted the measured dose outside of 7 cm. These differences are similar to those found in the MC‐calculated and measured dose profiles of the current clinical system as shown by Harris^2^ and Pitcher et al.^1^

### 3.C Out‐of‐field leakage dose (patient plane)

**Figure 6:**
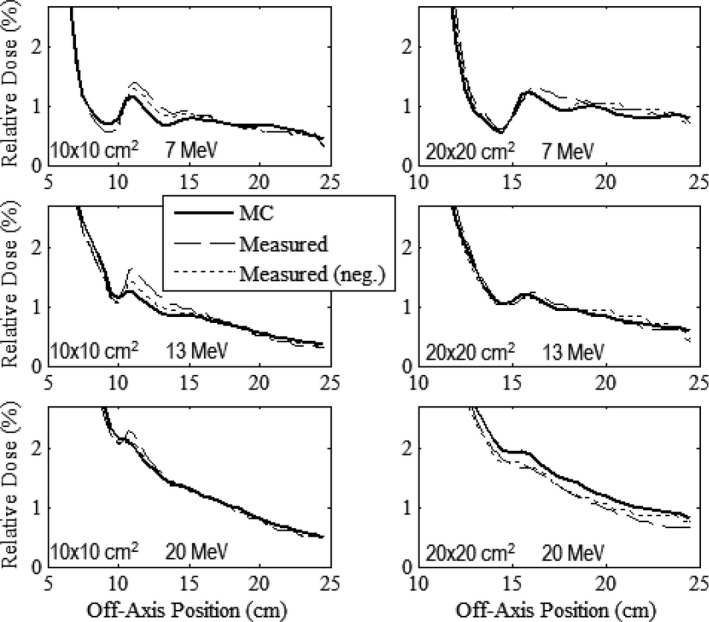
Comparison of measured and MC‐calculated in‐plane profiles of relative dose versus off‐axis position in water (100‐cm SSD) outside the field for the prototype 10 × 10 (left column) and 20 × 20‐cm^2^ (right column) applicators at beam energies of 7 (upper row), 13 (middle row), and 20 MeV (lower row). Profiles are normalized to central‐axis dose at the measurement depth, 1 cm in water. The long‐dashed curves represent the measured profiles. The short‐dashed curves represent the same measured profiles in the negative half plane, mirrored about central axis. The solid curve represents the MC‐calculated profile.

**Figure 7:**
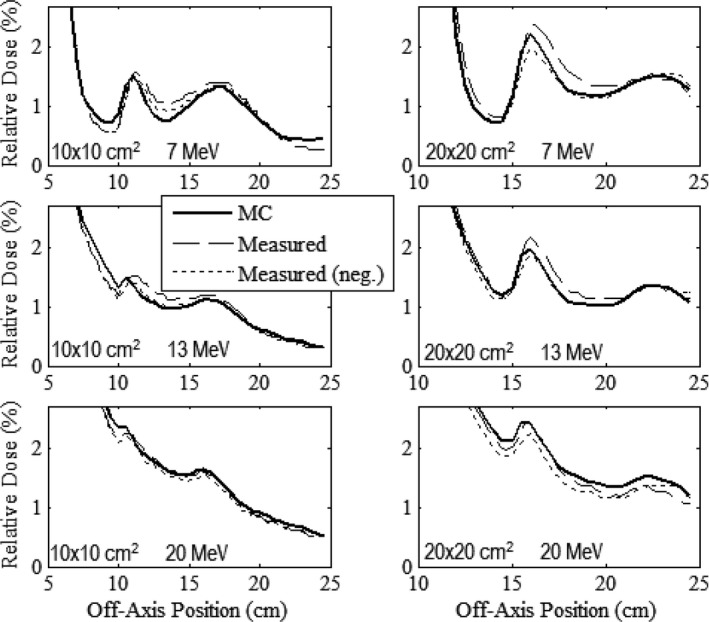
Comparison of measured and MC‐calculated cross‐plane profiles of relative dose versus off‐axis position outside the field with the same comparisons and measurement conditions as Fig. 6.

**Figure 8:**
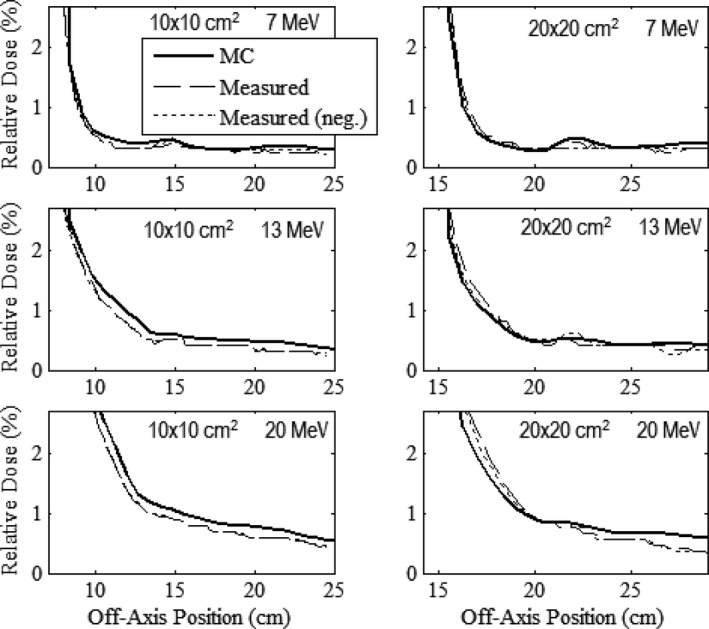
Comparison of measured and MC‐calculated positive diagonal profiles of relative dose versus off‐axis position outside the field with the same comparisons and measurement conditions as Fig. 6.

**Figure 9:**
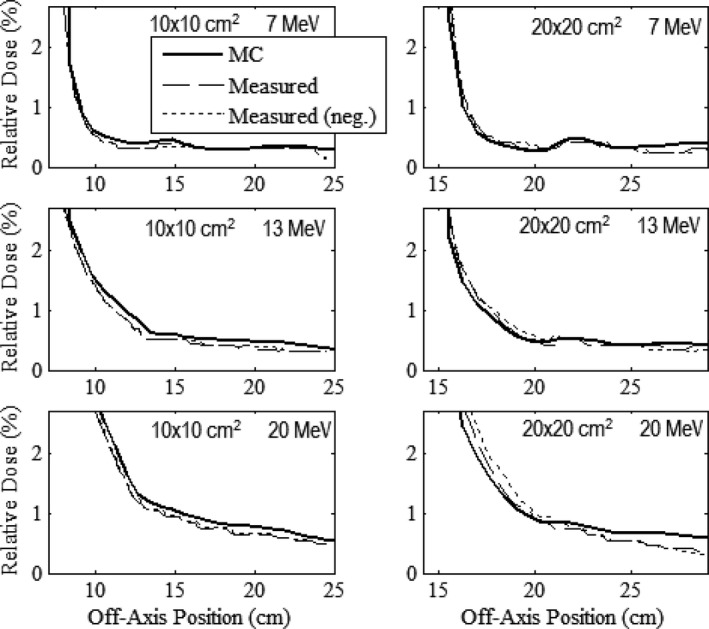
Comparison of measured and MC‐calculated negative diagonal profiles of relative dose versus off‐axis position outside the field with the same comparisons and measurement conditions as Fig. 6.

Figures 6, 7, 8, 9 compare measured and MC‐calculated out‐of‐field plots of relative dose versus off‐axis position for in‐plane, cross‐plane, positive diagonal, and negative diagonal axes, respectively. Each figure contains six plots, all combinations of the 10 × 10 and 20 × 20‐cm^2^ prototype collimation systems with the 7, 13, and 20 MeV beams. All profiles were measured at a depth of 1 cm in water at 100‐cm SSD. In general, MC calculations matched measured data to within 0.2% of central‐axis dose. For the major axes profiles of the 7 and 13 MeV beams, MC‐calculated data generally underpredicted measured data for both applicators, with a maximum difference of 0.39%. For the major axes profiles of the 20 MeV beam, MC‐calculated data generally slightly overpredicted measured data for both applicators, with a maximum difference of 0.21%. For the diagonal profiles, the MC‐calculated data generally overpredicted the measured data for all energies and applicators, with a maximum difference of 0.24%. The negative and positive measured profiles were shown to typically agree within 0.2% for all scans other than the cross‐plane profiles with the 20 × 20‐cm^2^ applicator, which had a maximum difference of 0.48% with the 7 MeV beam.

In general, both MC‐calculated and measured data show for all energies and applicators that cross‐plane profiles (Fig. 7) have greater leakage dose than in‐plane profiles (Fig. 6), presumed due to the increased scatter radiation from the X‐ray collimators in the cross‐plane direction.^1^ The diagonal profiles (Figs. 8 and 9) showed the least leakage dose, presumably due to their greater distance from central axis. Despite the potential benefit of fully understanding the physics of the leakage structure, from a clinical perspective, the important point is whether the applicators meet IEC leakage specifications. Therefore, the mean and maximum percent leakage doses determined from both the MC‐calculated and measured data for each beam energy and applicator investigated were calculated and are listed in Table 3.

**Table 3:** IEC‐specified mean (left columns) and maximum (right columns) leakage doses (percent of D_max_) determined from MC‐calculated and measured doses for the prototype collimation system (6 × 6–25 × 25 cm^2^) and 7, 13, and 20 MeV beams. Measured doses are shown only for the fabricated prototype applicators, 10 × 10 and 20 × 20 cm^2^. Bottom two rows show E_p,0_ values and maximum allowed leakage doses specified by the IEC for each nominal beam energy

Applicator size (cm^2^)	Dose calculation method	Mean percent leakage dose per IEC specifications			Maximum percent leakage dose per IEC specifications		
		7 MeV	13 MeV	20 MeV	7 MeV	13 MeV	20 MeV
6 × 6	MC calculated	0.45%	0.54%	0.91%	1.26%	2.17%	3.96%
	Measured	–	–	–	–	–	–
10 × 10	MC calculated	0.56%	0.63%	1.06%	1.53%	2.19%	3.93%
	Measured	0.57%	0.60%	0.99%	1.57%	2.04%	3.58%
14 × 14	MC calculated	0.68%	0.71%	1.16%	1.74%	2.00%	3.38%
	Measured	–	–	–	–	–	–
20 × 20	MC calculated	0.75%	0.76%	1.14%	2.22%	1.97%	2.86%
	Measured	0.76%	0.75%	1.01%	2.39%	2.16%	2.73%
25 × 25	MC calculated	0.74%	0.73%	1.06%	2.18%	1.95%	2.67%
	Measured	–	–	–	–	–	–
E_p,0_ (MeV)		7.14	13.12	20.47	7.14	13.12	20.47
IEC specified maximum		1.00%	1.10%	1.34%	10.00%	10.00%	10.00%

Both measured and MC‐calculated data in Table 3 show that the prototype applicators produced mean percent leakage doses well below IEC specifications for each beam energy and applicator combination. For example, the measured mean leakage dose at 7 MeV with the 20 × 20‐cm^2^ applicator had the closest value, being 0.24% below the 1.00% IEC specified maximum. For the MC‐calculated mean leakage doses, the 20 MeV beam with the 14 × 14‐cm^2^ applicator had the closest value, being 0.18% below the 1.34% IEC specified maximum. Similarly, data in Table 3 show that the prototype applicators produced maximum percent leakage dose well below the IEC specified value of 10.00% of D_max_ for all beam energies. For the measured maximum percent leakage doses, the 20 MeV beam with the 10 × 10‐cm^2^ applicator had the closest value, being 3.58%. For the MC‐calculated maximum percent leakage dose, the 20 MeV beam with the 6 × 6‐cm^2^ applicator had the closest value, being 3.96%.

Table 4 compares the mean and maximum percent leakage doses, both measured and MC‐calculated values, of the prototype 10 × 10 and 20 × 20‐cm^2^ applicators with those of the current clinical applicators for the 7, 13, and 20 MeV beams. The MC‐calculated and measured values agree within 0.1% in most cases and show similar trends. For mean percent leakage doses, the 20 × 20‐cm^2^ prototype applicator shows slightly (<0.2%) greater values for all energies; the 10 × 10‐cm^2^ prototype applicator shows slightly (<0.1%) less values for the 7 and 13 MeV beams and slightly (<0.1%) greater values for the 20 MeV beam. These differences are small, and the prototype applicators comfortably meet IEC mean leakage dose specifications.

**Table 4:** MC‐calculated (upper) and measured (lower) mean and maximum leakage doses (percent of D_max_) for the prototype 10 × 10 and 20 × 20‐cm^2^ applicators are compared with those for the current clinical Elekta applicators (MLCi2 treatment head) for the 7, 13, and 20 MeV beams

Applicator size (cm^2^)	Applicator design	Mean percent leakage dose			Maximum percent leakage dose		
		7 MeV	13 MeV	20 MeV	7 MeV	13 MeV	20 MeV
MC‐calculated dose							
10 × 10	Current	0.70%	0.65%	0.99%	2.11%	2.75%	4.38%
	Prototype	0.56%	0.63%	1.06%	1.53%	2.19%	3.93%
20 × 20	Current	0.67%	0.69%	0.93%	1.65%	1.95%	2.83%
	Prototype	0.75%	0.76%	1.14%	2.22%	1.97%	2.86%
Measured dose							
10 × 10	Current	0.66%	0.67%	0.93%	2.14%	2.78%	4.09%
	Prototype	0.57%	0.60%	0.99%	1.57%	2.04%	3.58%
20 × 20	Current	0.56%	0.65%	0.85%	1.74%	2.03%	2.73%
	Prototype	0.76%	0.75%	1.01%	2.39%	2.16%	2.73%

For maximum percent leakage dose, the 20 × 20‐cm^2^ prototype applicator shows similar values at 20 MeV, slightly (<0.15%) greater values at 13 MeV, and 0.65% greater values at 7 MeV. The 10 × 10‐cm^2^ prototype applicator shows much smaller values (0.51%–0.74%) for the 7, 13, and 20 MeV beams. Nonetheless, the maximum percent leakage dose values for all current and prototype applicators remain well below the IEC allowance of 10%.

### 3.D Out‐of‐field leakage dose (lateral to trimmers)

**Figure 10:**
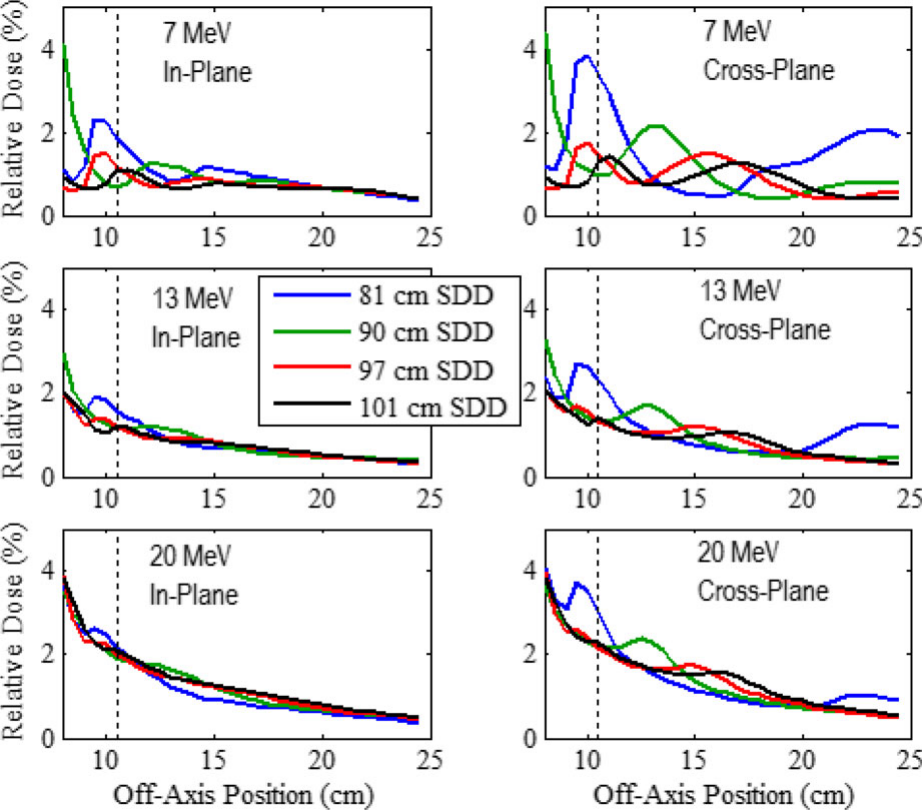
Leakage dose lateral to prototype 10 × 10‐cm^2^ applicator. Profiles of MC‐calculated relative dose versus off‐axis position for the 7, 13, and 20 MeV beams. In‐plane (left column) and cross‐plane (right column) leakage relative dose profiles are at *z* positions of 81 (blue), 90 (green), 97 (red), and 101 (black) cm. All profiles were calculated at 1‐cm depth in water and normalized to D_max_ at 100‐cm SSD. The vertical dashed line represents the off‐axis position 2 cm outside the outer edge of the middle trimmer, outside which IEC specifies the dose not to exceed 10%.

**Figure 11:**
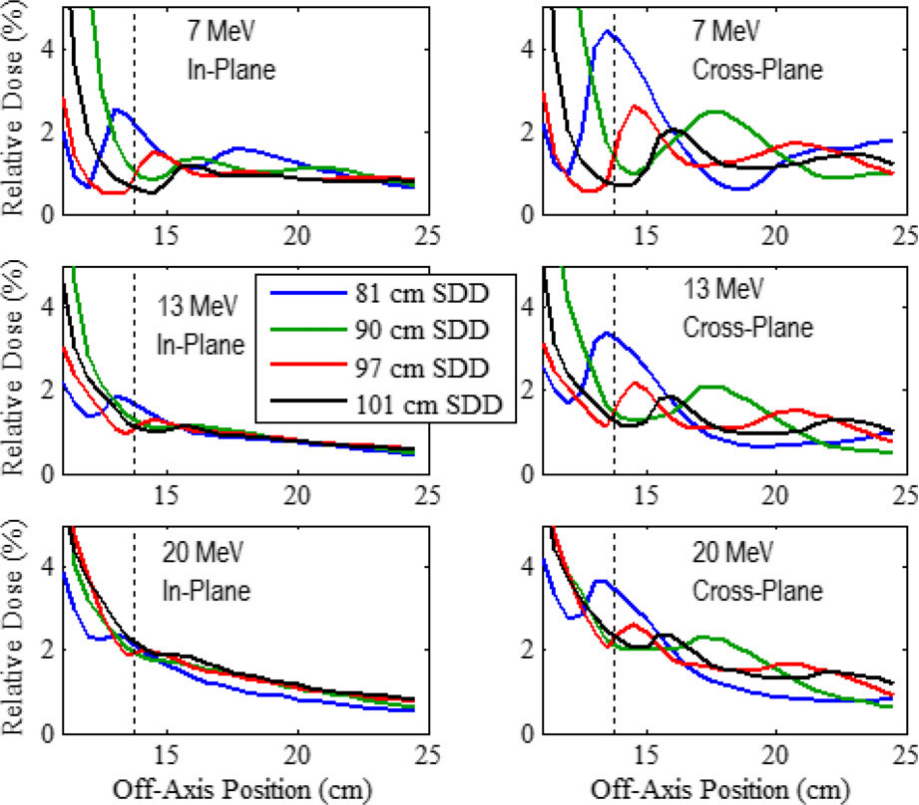
Leakage dose lateral to prototype 20 × 20‐cm^2^ applicator. Profiles of MC‐calculated relative dose versus off‐axis position at *z* positions of 81 (blue), 90 (green), 97 (red), and 101 (black) cm with the same comparisons and measurement conditions as Fig. 10.

Along with the criteria for mean and maximum leakage in the patient plane, the IEC specifies that the maximum dose along the side of the applicator should not exceed 10% of D_max_, measured 2 cm outside the volume contained by the applicator. For assessment of this specification, results of our MC calculations outside the prototype 10 × 10 and 20 × 20‐cm^2^ applicators for the 7, 13, and 20 MeV beams are shown in Figs. 10 and 11, respectively, where in‐plane and cross‐plane profiles of relative dose versus off‐axis position are plotted for *z* positions of 81, 90, and 97 cm and the patient plane (*z *=* *101 cm). All profiles were calculated at 1‐cm depth in water and normalized to D_max_ at 100‐cm SSD. The vertical dashed line in each plot represents the off‐axis position 2 cm outside the outer edge of the middle trimmer (at off‐axis positions of 10.5 and 13.8 cm for the 10 × 10 and 20 × 20‐cm^2^ applicators, respectively).

The calculated profiles all have three common characteristics: (a) there is a general trend of the leakage dose to decrease with increasing distance from the applicator; (b) exceptions to (a) are characterized by peaks, which are believed based on electrons scattered from inner edges of the electron collimating system (trimmers and x‐ray collimators),^1^ (c) in‐plane is less than cross‐plane leakage; and (d) leakage doses in the plots for all cases were well below the IEC specification of 10%, the maximum being 4.29% occurring in the 7 MeV cross‐plane profile with the 20 × 20‐cm^2^ applicator calculated at a *z* position of 81 cm. These results imply that there is no leakage 2 cm outside the trimmer volume that exceeds 10% and that the newly designed applicators produce acceptably low leakage along their sides.

## 4 CONCLUSIONS

Results of this study showed that measurements using prototype 10 × 10 and 20 × 20‐cm^2^ applicators agreed with calculated weights, in‐field flatness, and out‐of‐field leakage doses for Pitcher et al.'s 5 design of a new Elekta electron collimating system for 7, 13, and 20 MeV beams. Also, MC‐calculated results showed that in‐field flatness and out‐of‐field leakage doses for all applicators (6 × 6–25 × 25‐cm^2^) and 7, 13, and 20 MeV beams met our stringent flatness and IEC leakage dose specifications, respectively. Hence, we conclude that our results validated the new Elekta electron collimating system designed by Pitcher et al.^5^ for 6–20 MeV electron beams. This design should serve well as the basis for a new clinical electron collimating system with significantly reduced applicator weights.

## CONFLICTS OF INTEREST

This research was funded in part through a research agreement with Elekta Limited.
